# Prohibitin: targeting peptide coupled to ovarian cancer, luteinization and TGF-β pathways

**DOI:** 10.1186/s13048-017-0325-4

**Published:** 2017-04-20

**Authors:** Nour M. El-Etreby, Amany A. Ghazy, Radwaa Rashad

**Affiliations:** 1 Department of Obstetrics and gynecology, Faculty of Medicine, Alexandria University, Alexandria, Egypt; 2 Department of Microbiology and Immunology, Faculty of Medicine, Kafrelskeikh University, Kafrelskeikh, Egypt; 3 Department of Pathology, MedicalResearch Institute, Alexandria University, Alexandria, Egypt

**Keywords:** Ovarian epithelial tumor, Prohibitin, LH, LHRmRNA, TGF-β

## Abstract

**Background:**

Ovarian epithelial tumor (OET) is a silent disease of late diagnosis and poor prognosis. Currently treatment options are limited and patient response to treatment is difficult to predict so there is a serious need to delineate the real pathogenesis to predict tumour prognosis. Prohibitin (PHB) is an evolutionarily protein that regulates the cell cycle. TGF-β has been shown to be a positive and negative regulator of cellular proliferation and differentiation.

The present study provides an overview on the role played by PHB1, TGF-β and LH in ovarian cancer.

**Methods:**

The study was conducted on 60 patients with ovarian tumors (benign, borderline and malignant) and 20 healthy volunteers. LH and TGF-β serum levels were measured by ELISA. Expression of prohibitin and LHR-mRNA were assessed by IHC and TaqMan® real time gene expression assay, respectively.

**Results:**

Serum levels of LH and TGF-β were significantly decreased among borderline and malignant groups. There was significant over-expression of LHRmRNA in malignant group. Prohibitin expression was significantly increased in malignant ovarian tissue. Strong negative correlations were found between LHR mRNA expression and serum LH levels, and between IHC score of prohibitin and serum levels of LH among patients with borderline ovarian tumors.

**Conclusion:**

Steady decline of LH and TGF-B serum levels, from benign cystadenoma to borderline tumor to carcinoma, suggests their inhibitory role against OET cell growth. Increased PHB1 expression in OET suggests its proliferative activity that can be regulated by luteinisation and/or TGF-β. Furthermore increased LHR mRNA tissue expression can provide hope for using LH in treatment of some types of ovarian cancers.

## Background

Ovarian epithelial tumor (OET) is a silent disease of usually late diagnosis and poor prognosis. Currently treatment options are limited and patients’ response to treatment is difficult to predict and there is a serious need for anticipating tumour response and orientating medical choices [1].

Prohibitins (PHB), a highly conserved group of proteins, are ubiquitously expressed in many cell types and are mainly located in the mitochondria, nucleus and plasma membrane. PHB1 and PHB2 are two highly homologous subunits composed of N-terminal trans-membrane domain and C-terminal coiled-coil domain that is involved in protein- protein interactions as well as transcriptional regulation. At the plasma membrane, PHB is a trans-membrane adaptor that activates downstream signal transduction [2]. The appropriate distribution of PHB1 between the nucleus and cytoplasm is maintained by a dynamic balance between the nuclear import and export signals in response to specific signals as well as its ability to differentially regulate the transcriptional activity of E2F (survival) and P53 (apoptotic) signals [3].

OET is mainly derived from the ovarian surface epithelium (OSE) resulting from aggregation of the epithelial cells within the stroma resulting in the formation of inclusion cyst. OSE is a stationary mesothelium (share a common embryological origin to peritoneum) which retains the capacity to alter state of differentiation along stromal and epithelial phenotypes. They alter the motility and proliferative response through inducers such as epithelial growth factor (EGF), collagen and transforming growth factor beta (TGF-β) [4].

Prohibitin gene is located on chromosome 17q21 close to the ovarian and breast carcinoma susceptibility gene (BRCA1) locus and may function as growth regulatory molecule within several tissues, including the ovary [5]. Further evidence suggesting that high PHB may function as growth regulatory molecule and is activated upon gonadotropin stimulation is. They suggested that luteinizing hormone (LH) might be inhibitory for OET cell growth [6]. Thompson et al. [7] have stated that PHB1 is associated with mitochondrial destabilization in ovarian cancer.

TGF-β1 is a polypeptide homodimer that was first discovered by its ability to induce transformation in normal cells [8]. Zheng et al. [3] stated that the nuclear import and export signals of PHB1 in response to specific signals as well as its ability to differentially regulate the transcriptional activity of E2F (survival) and P53 (apoptotic) signals is maintained by a dynamic balance. They have suggested regulation of PHB1 by TGF-B1 through different signalling pathways in prostate cancer. This agrees with Zhu et al. [9] who have suggested that TGF-B regulates PHB1 through smad-dependent pathway (apoptosis) and MAPK intra-cellular/survival pathway.

In the present work, we measured the levels of LH and TGF-β1 in serum and evaluated the tissue expression of PHB1 and LH mRNA in benign, borderline and malignant human ovarian tumors to declare their roles in tumorgenesis.

## Subjects

This study was conducted on 80 subjects; 60 patients with ovarian tumors selected from Obstetrics and gynaecology department, Faculty of Medicine, Alexandria University and 20 healthy volunteers. Subjects were divided into 4 groups; group 1 included 20 patients with benign lesions (7 at menopause while 13 in pre-menopause state), group 2 included 20 patients with borderline ovarian cancer (12 at menopause while 8 in pre-menopause state), group 3 included 20 patients with malignant ovarian cancer (10 at menopause and 10 in pre-menopause state), and group 4 (control group) included age matched healthy females (10 at menopause and 10 in pre-menopause).

The study protocol was approved by the ethical committee of Faculty of Medicine, Kafrelsheikh University. In addition, the study followed the ethical guidelines of the Faculty of Medicine, Alexandria University as all participants were asked to freely volunteer to the study and informed written consents were collected from them prior to their inclusion in the study (Informed Written Consent for Patient Participation in a Clinical Research, 2011).

Inclusion criteria of patients include females aged between 30 and 60 years old suffering from different types of ovarian tumors to be grouped later. Exclusion criteria include ages below 30 or over 60 years, any autoimmune disease, any infectious disease or any other tumor that may affect the results of the study parameters.

## Methods

All reagents used in the study were supplied by Applied Biosystems, Life Technology Company (St. Louis, MO, USA) and Sigma–Aldrich Chemical (St. Louis, MO, USA) unless otherwise stated.

1. Quantitative determination of LH in serum
  Direct quantitative determination of LH in serum samples was done by commercial LH ELISA kit (11-LUTHU-E01, ALPCO, United States of America), following the manufacturers’ instructions. The results were expressed in IU/L.
2. Measurement of TGF-β serum levels:
  TGF-β levels in serum samples were measured using Human TGF-beta Platinum ELISA kit (BMS254, eBioscience, Germany), according to the manufacturers’ protocol. All standards, controls and patient samples were performed in triplicates and the average absorbance values for each were calculated. Using semi-logarithmic graph paper, a standard curve was constructed by plotting the mean absorbance obtained from each standard against its concentration with absorbance value on the vertical (Y) axis and concentration on the horizontal (X) axis. Using the mean absorbance value for each sample the corresponding concentration from the standard curve was determined. The concentration of TGF-β in each sample was multiplied by the initial dilution factor. Levels were expressed by pg/ml.
3. Pathologic features of studied cases
  Hematoxylin and Eosin (H&E)-stained slides from a total of 80 cases were reviewed, characterized and studied. All malignant cases were diagnosed and graded using criteria of the International Federation of Obstetrics and Gynecology (FIGO) [10].
4. Evaluation of LHR mRNA expression
  Assessment of LHR mRNA expression was performed using *StepOne*™ Real-Time PCR System, following the instructions in TaqMan® Gene Expression Assays protocol provided from Applied Biosystems.
  Genomic RNA was extracted from ovarian tissue samples using the PureLink® RNA Mini Kit (12183018A, 12183025, Ambion, Life Technologies) followed by assessment of RNA concentration and purity using a Nanodrop spectrophotometer. All RNA samples had an optical density OD260:OD280 ratio of between 1.8 and 2.0. RNA samples were used immediately in reverse transcription.
  Reverse transcription of RNA to complementary DNA (cDNA) was carried out using high capacity cDNA reverse transcription kit (Applied Biosystems, Life Technology Company) according to the manufacturers’ protocol. All pipetting steps were done on ice.
  Quantitative RT-PCR was performed in StepOne™ Real-Time PCR System (Applied Biosystems, Life Technologies) using TaqMan® Gene Expression LHR Assays (TaqMan® MGB probes, FAM™ dye-labeled) following the manufacturers’ instructions. The results were calculated according to the manufacturers’ instructions as follow: ∆CT = CT of LHR- CT of house-keeping gene, ∆∆CT = ∆CT of patient-mean of ∆CT of LHR, relative quantity (RQ) = 2^-∆∆CT^, RQ < 1 (i.e., control) means under-expression, RQ > 1 means over-expression,
5. Assessment of prohibitin expression
  Immunohistochemical (IHC) stains were performed on 5-μm tissue sections from representative blocks of ovarian tissue using the purified mouse anti-prohibitin antibody (E-5): sc-377037 (mAb provided by Santa Cruz Biotechnology) and the standard avidin-biotin-complex technique as described previously [3, 11].
  Evaluation of results as positive or negative was one as described previously [12]. The percentage of neoplastic cells and non-neoplastic tissues that showed dark brown cytoplasmic staining was recorded. The staining intensity was graded for both on the following scale: 0 = no staining; 1 = weak staining; 2 = moderate staining; and 3 = intense staining. Prohibitin IHC index was generated by multiplying the intensity by the percentage of positive cells in a defined specimen, yielding scores ranging from 0 to 300. Stained cells exceeding 10% were considered positive and staining was performed in duplicate.

### Statistical analysis of the data

Data were analyzed using IBM SPSS software package version 20.0. Qualitative data were described using number and percent. Comparison between different groups regarding categorical variables was tested by Chi-square test. F-test (ANOVA) for normally quantitative variables, was used to compare between more than two studied groups, and Kruskal Wallis test for abnormally quantitative variables, to compare between more than two studied groups, and Spearman coefficient test to correlate between variables [13].

## Results

I. Subject’s demographic data:
  Age distributions among benign, borderline and malignant ovarian tumors and healthy control group were ranged between 40 and 60 years old. There weren’t any statistically significant differences between the studied groups regarding age or menstrual state.
II. Serum levels of LH among studied groups:
  The means of serum levels of LH among studied groups were summarized in Table 1. There was statistically significant decrease in its levels among patients with borderline and malignant ovarian tumors (*p* = 0.001*). Furthermore, there was statistically significant increase in LH serum levels among patients with benign lesions when compared to the other studied groups (*p* < 0.001*).

**Table 1:** Comparison between the studied groups regarding serum levels of LH & TGF-B

Parameters	Group				F (P)
	Control	Benign	Borderline	Malignant	
LH (Mean ± SD)	8.7 ± 1.6	18.8 ^d^ ± 4.4	4.5 ± 0.9	3.7 ± 1.4	0.001*
TGF-B (Mean ± SD)	1347.4 ± 594.2	1039.0 ± 686.4	994 ± 547.6	894.0 ± 547.6	0.065

**Fig. 1:**
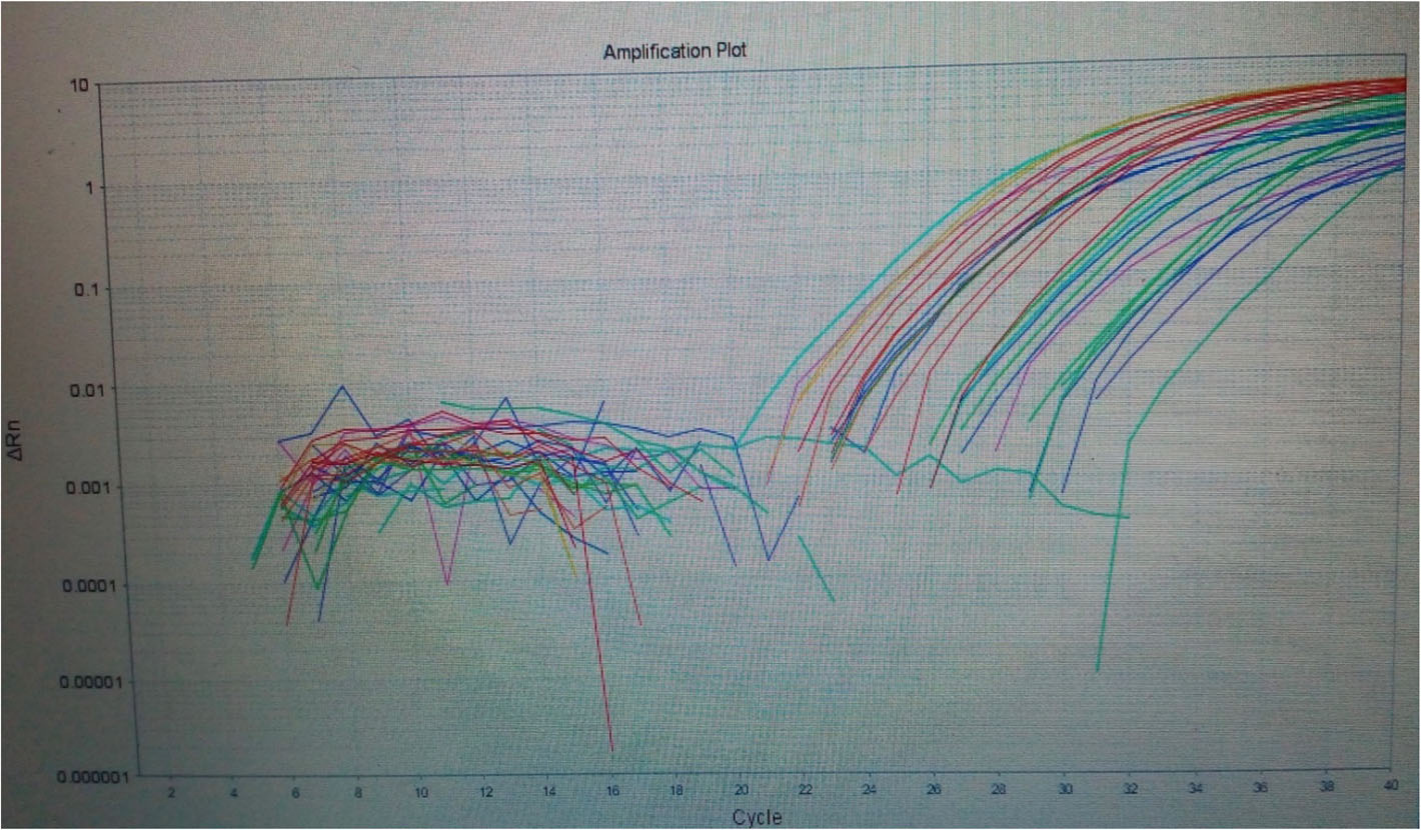
RT-PCR amplification blot showing expression of LHRmRNA and GAPDH in the studied groups

*F* One Way ANOVA, *d* significantly different group (s), *P < 0.05 (significant)
III. Serum levels of TGF-β among studied groups:
  The means of serum levels of TGF-β were summarized in Table 1. There was statistically non-significant decrease in its levels with progression of ovarian tumors (*p* = 0.065).
IV. Pathologic features of studied cases
  H&E-staining of slides from a total of 80 cases have clarified that the benign ovarian lesions were 12 serous and 8 mucinous cystadenomas. Borderline lesions were 15 serous and 5 mucinous borderline tumors. The cases of ovarian carcinomas were serous (*n* = 9), mucinous (*n* = 4), endometrioid (*n* = 5), and poorly or undifferentiated (*n* = 2). The mucinous carcinomas were all ovarian primary by clinicopathologic studies.
V. LHRmRNA expression in ovarian tissue:
  The relative quantitation of LHRmRNA expression among studied groups showed statistically significant over-expression of LHRmRNA among patients with malignant tumors (*p* = 0.001*) (Fig. 1, Table 2). There was strong negative correlation between LHR mRNA expression and serum levels of LH among studied groups.

**Table 2:** Comparison between the studied groups regarding LHRmRNA tissue expression

RQ of LHRmRNA	Group				^H^ P
	Control	Benign	Borderline	Malignant	
• Mean	0.065	1.426	19.998	25.047	0.001*
• SD	0.073	0.900	31.066	53.626	

**Fig. 2:**
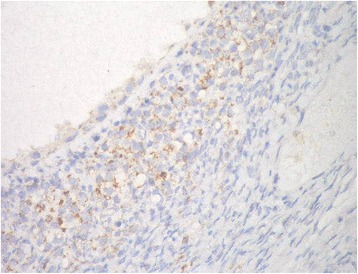
Prohibtin expression in normal ovarian tissue showing mild cytoplasmic staining of the luteinized cells lining follicular cyst (IHC; × 40)

**Fig. 3:**
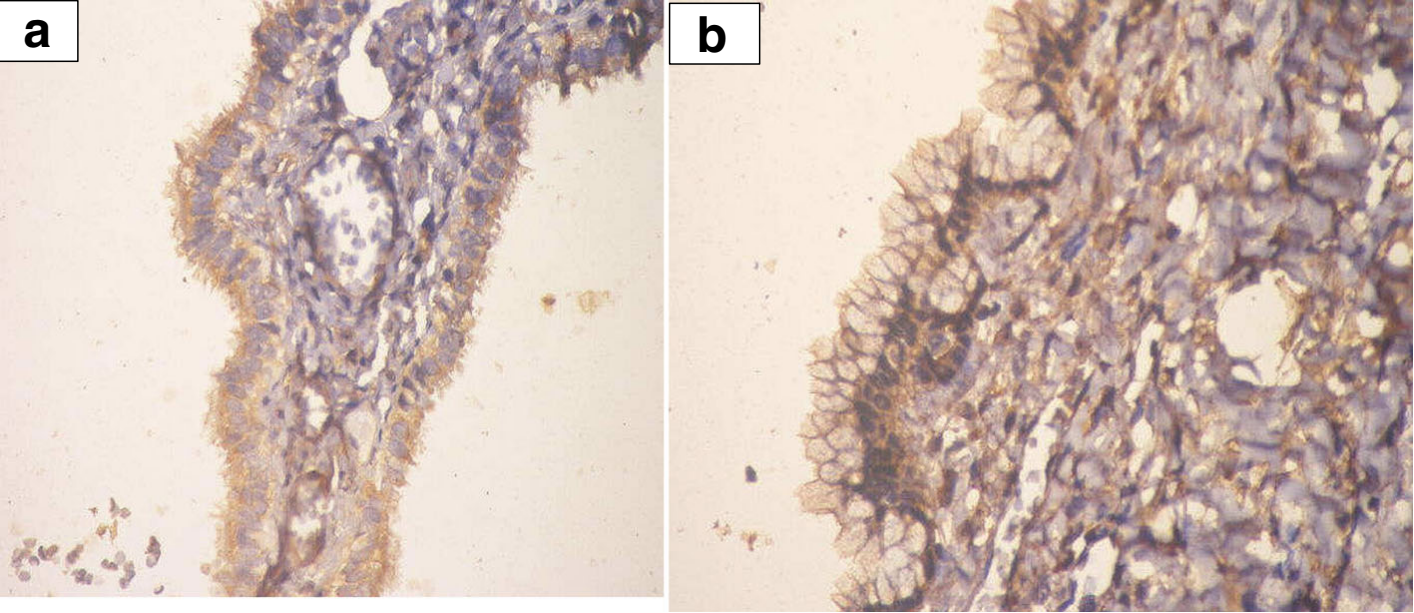
**a** Prohibtin expression in benign papillary serous cystadenoma. (IHC; ×40). **b** Prohibitin expression in benign mucinous cystadenoma.(IHC; ×40)

**Fig. 4:**
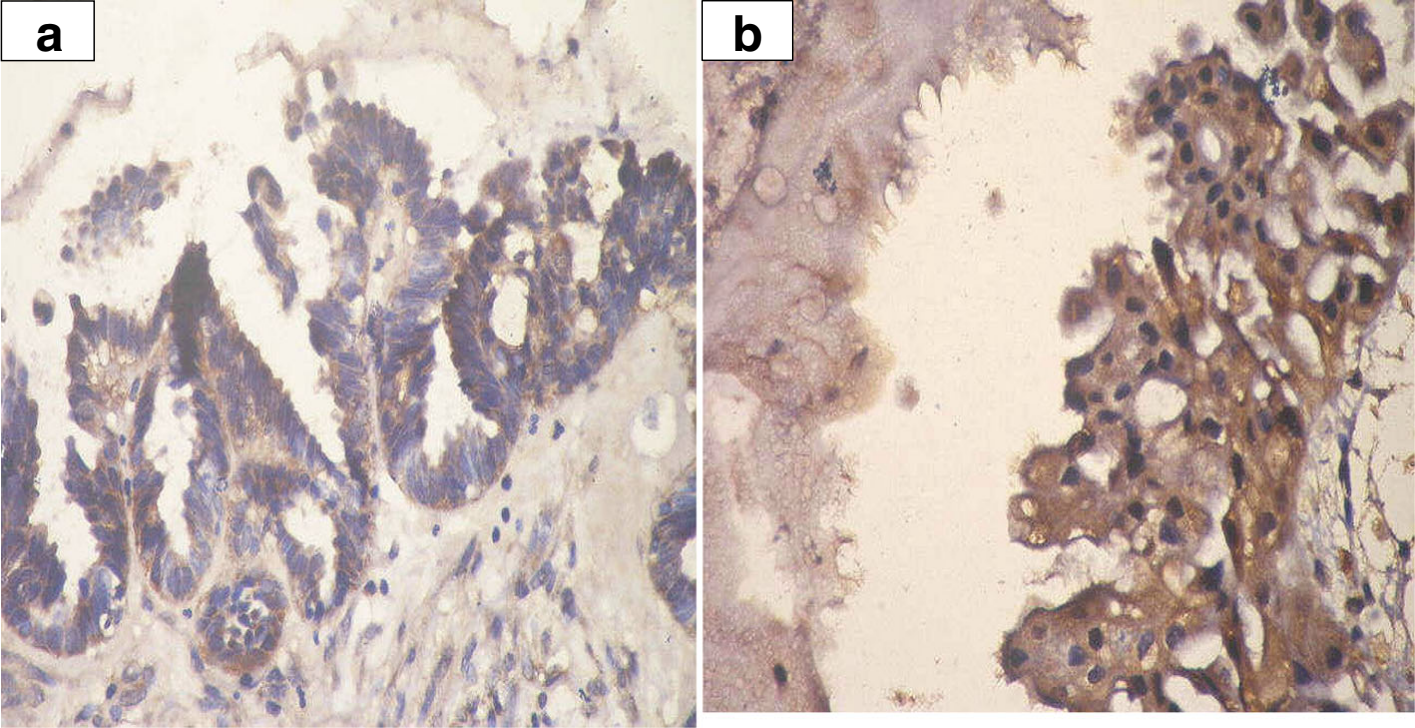
**a** Prohibitin expression in borderline serous tumor. (IHC; ×40). **b** Expression of prohibitin in borderline mucinous tumor. (IHC; ×40)

**Fig. 5:**
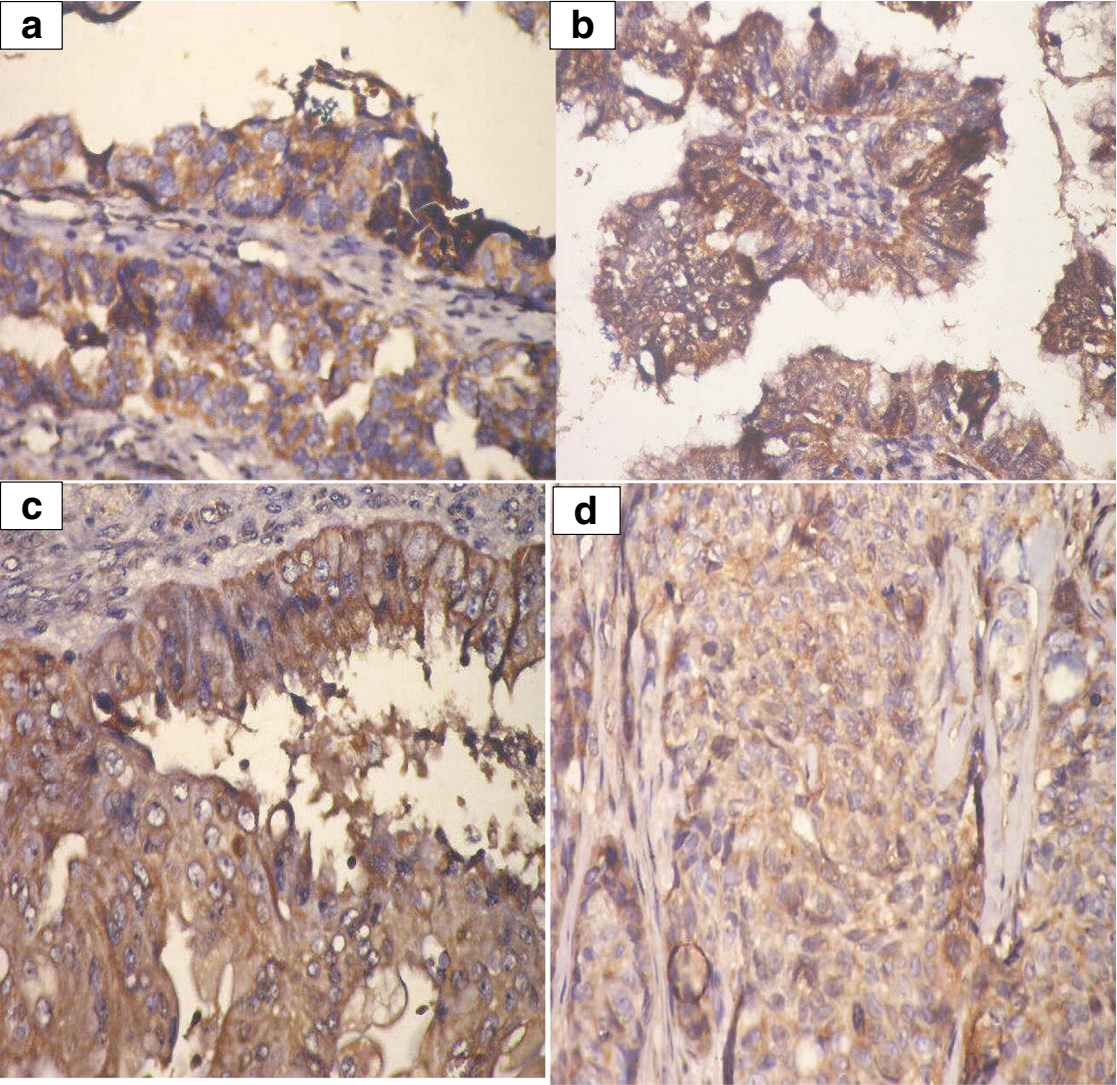
**a** Prohibitin expression in malignant papillary serous cystadenocarcinoma. (IHC; ×40). **b** Prohibitin expression in malignant mucinous cystadenocacinoma. (IHC; ×40). **c** Prohibitin expression in malignant endometrioid carcinoma (IHC; ×40). **d** Prohibitin expression in poorly differentiated carcinoma (IHC; ×40)

*RQ* relative quantification, *H* Kruskal-Wallis test, *P < 0.05 (significant)
VI. Prohibitin expression in ovarian tissue:
  IHC was employed to determine the expression levels of prohibitin in physiological ovarian tissue and ovarian epithelial tumors, and the immunostaining scores are summarized in Table 3. Control ovarian tissue section from follicular cyst showed mild cytoplasmic staining of the luteinized cells lining the cyst (Fig. 2). Figures 3a & b represent prohibitin expression in benign papillary serous and mucinous ovarian tumors, respectively. Figures 4a, b & c represent prohibitin expression in borderline papillary serous and mucinous ovarian tumors, respectively. Figures 5a, b, c & d represent prohibitin expression in malignant papillary serous, mucinous, endometrioid and undifferentiated ovarian tumors, respectively. There was a statistically significant increase of prohibitin expression with progression from benign to malignant tumors (*P* < 0.001*). IHC score of prohibitin showed a strong negative correlation with serum levels of LH among patients with borderline ovarian tumors.

**Table 3:** Comparison between the studied groups regarding IHC expression of prohibitin

Parameters	Group				F (P)
	Control	Benign	Borderline	Malignant	
Intensity (Mean ± SD)	2.2 ± 0.6	2.0 ± 0.0	2.6 ^d^ ±0.5	3.0 ^d^ ±0.0	0.001*
% (Mean ± SD)	69.3 ± 10.2	66.3 ± 16.0	85.6 ^d^ ±9.0	96.3 ^d^ ±3.9	0.001*
Score (Mean ± SD)	147.8 ± 43.5	132.5 ± 31.9	226.7 ^d^ ±64.4	288.8 ^d^ ±11.8	0.001*

*F* One Way ANOVA, *d* significantly different group (s), *P < 0.05 (significant)

## Discussion

There are contradictory findings concerning the role of PHB in cancer cell survival. Results of the present work suggest that PHB1 expression, accompanied by decreased LH in serum, increased LHR mRNA expression in ovarian tissue and decreased TGF-B serum levels, may contribute to the proliferative activity of ovarian cancer cells. These results are supported by Gregory-Bass et al. [14]; Sanchez-Quiles et al. [15], Zhu et al. [9]; Wu and Wu [16], Zhou and Qin [17]; and Zhang et al. [18].

Gregory-Bass et al. [14] have proved that over-expression of PHB1 by adenoviral PHB1 infection resulted in an increase in the percentage of ovarian cancer cells accumulating at G0/G1 phase of the cell cycle promoting survival of cancer cells. Moreover, they found that PHB1 over-expression induce cellular resistance to chemotherapy by decreasing the sensitivity of cancer cells to apoptosis. They suggested PHB1 as a possible candidate protein that contributes to development of drug resistance in OET.

Some studies have stated that knockdown or block of PHB result in enhancement of apoptosis in human hepatoma cells [15], ultraviolet B light-irradiated HaCaT keratinocytes [16], gastric carcinoma cell line SGC7901 [18], and bladder cancer [19]. Zhou and Qin [17] have reported that prohibitin expression was increased in most of the cancers and its signaling pathways might be very important in the pathogenesis of diseases.

Additionally, Zhu et al. [9] have investigated PHB involvement in the survival and/or apoptostic outcomes of human prostate cancer cell in response to TGF-β. Their calcein-based immunofluorescence studies have revealed that PHB has a functional role in maintaining inner mitochondrial membrane permeability, and increased cell survival. Loss of PHB function in prostate cancer cells led to enhanced apoptotic response to TGF-β. They stated that in the early stage TGF-β acts as a tumor suppressor through smad 2/3 and smad 1/5. However during cancer progression, TGF-β bypassing the Smads and activates Raf-MEK signaling causing recruitment and phosphorylation of PHB leading to increased cell survival and invasion. These findings suggest a dual role for PHB as a downstream determinant of the cellular response to TGF-β via Smad-dependent pathway (apoptosis) and MAPK intracellular signaling (survival). These results showed strong link between PHB and TGF-B and provide evidence that different signalling pathways in the different kinds of cells regulates the relation between both parameter resulting in inhibition or enhancement of tumor growth.

Thuaud et al. [20] have documented that phosphorylation modulates both the subcellular localization of PHBs and their downstream effects on proliferation and survival of cells.

Peng et al. [2] have provided another explanation for the different roles of PHB in regulating the cellular functions. They suggested that these roles are determined by the subcellular localization of PHB where membrane prohibitin regulate the membrane transport signaling, nuclear PHB control the cell cycle, and mitochondrial prohibitin modulates mitochondrial dynamics, and the mitochondrial induced intrinsic apoptotic pathway. Consequently, any alteration in PHB expression or location may influence cell fate regarding the regulation of cell survival and apoptosis.

Li [21] has reported that “TGFB superfamily signaling regulates essential female reproductive processes and is indispensable for ovarian development and function. Dysregulation of TGFB signaling results in cellular and molecular deficiencies in the ovary, leading to reproductive diseases and cancer development.

On contrary to our results, Jia et al. [12] have studied the role of LH in OET development. They analyzed total proteins from OET cells treated with gonadotropins by proteomics and detected prohibitin expression in the serous tumors. They found that PHB is upregulated by LH and there was a steady decrease prohibitin expression from benign serous cystadenomas to serous carcinomas. They suggested that PHB is protective from ovarian cancer development and progression, and LH may play an inhibitory role in ovarian tumorigenesis.

## Conclusion

Steady decline of LH and TGF-B serum levels, from benign cystadenoma to borderline tumor to carcinoma, suggests their inhibitory role against OET cell growth. Increased PHB1 tissue expression can suggest its proliferative activity during ovarian carcinogenesis. We suggest PHB1 expression can be regulated by luteinisation and/or TGF-β, thus ovarian oncogenesis can be aborted. Furthermore increased LHR mRNA tissue expression can provide hope for using LH in treatment of some types of ovarian cancers. Additional studies are required to decipher the efficacy of administration of LH analogues or TGF-β in decreasing PHB expression by OET and reversing ovarian oncogenesis.

## Abbreviations

FIGO: International federation of obstetrics and gynecology
IHC: Immunohistochemical stain
LH: luteinizing hormone
OET: Ovarian epithelial tumor
OSE: Ovarian surface epithelium
PHB: Prohibitin
TGF-β: Transforming growth factor beta
